# Electrical, optical, and electrochemical performances of phosphate-glasses-doped with ZnO and CuO and their composite with polyaniline

**DOI:** 10.1038/s41598-023-51065-5

**Published:** 2024-01-12

**Authors:** Ragab Mahani, A. Kh. Helmy, A. M. Fathi

**Affiliations:** 1 Microwave Physics and Dielectrics Department, National Research Centre, 33 El Bohouth St. (Former El Tahrir St.), P.O. 12622, Giza, Egypt; 2 Glass Research Department, National Research Centre, 33 El Bohouth St. (Former El Tahrir St.), P.O. 12622, Giza, Egypt; 3 Physical Chemistry Department, National Research Centre, 33 El Bohouth St. (Former El Tahrir St.), P.O. 12622, Giza, Egypt

**Keywords:** Chemistry, Energy science and technology, Materials science, Physics

## Abstract

Phosphate-based glasses (PBG) with appropriate doping agents have been used as solid electrolytes in solid-state ionic devices. Therefore, more light was shed on the electrical, optical, and electrochemical behavior of the phosphate-based glasses (PBG), containing ZnO or CuO in the absence and existence of conductive polyaniline (PANI), since no publications are available concerning this work. The glass samples were prepared by the rapid quenching method, then mixing phosphate glass and polyaniline (PANI) with metal oxide (ZnO, CuO). They were characterized by different techniques; diffuse reflectance spectrophotometer (DRS), broadband dielectric spectrometer (BDS), cyclic voltammetry (CV), and charge–discharge techniques. In the DRS study, the direct and indirect band gap were calculated from Tauc’s relationship where CuO-doped glasses have higher values than ZnO-doped glasses. In the BDS study, the permittivity of all glass compositions decreased while AC conductivity increased with increasing frequency. AC conductivity of PBG doped with metal oxides and mixed with PANI exhibited semiconducting features (6.8 × 10^–4^ S/cm). Further, these composites exhibited lower loss tangent (0.11), and giant permittivity (186,000) compared to the pure PBG. Also, the electrochemical study exhibited that the composite with 7% CuO content has the highest specific capacitance value (82.3 F/g at 1.0 A/g) which increased to about 113% of its first cycle and then decreased to about 55% after 2500 cycles and finally increased again to 77% after 4500 cycles, indicating its good stability. The combination of optical, electrical, and electrochemical features of these composites suggests their use for energy generation and storage devices.

## Introduction

As energy is the first demand for major world powers, researchers have an interest in developing and producing materials that are used as clean, lightweight energy storage materials. Therefore, Li-ion batteries (LIBs), and supercapacitors are “superstars” in the investigation fields^1–4^. Long time for discharge, short time for charging and high capacity for storage are from the suggested properties of these materials^4^. Different materials have been explored to achieve some of these properties like polymers, glasses, ceramics, glass ceramics, and their composites. High breakdown strength of polymers (> 5 MV/cm) despite relatively low *ε*_*r*_ (< 5) suggest their use for capacitors^5^. Particularly, Poly (3,4-ethylene dioxythiophene) (PEDOT), polyaniline (PANi), and polypyrrole (PPy) as conducting polymers (CPs), have been considered as good materials for energy storage devices since the discovery in 1960^6^ because of their high conductivity, high specific capacitance, faster kinetics than most inorganic batteries^7,8^. In addition, glass materials have high breakdown strength and low *ε*_*r*_^9^, since many tries have been made to raise *ε*_*r*_ of the glass without changing its high breakdown strength. Different properties of phosphate glasses make their use in technological purposes easy such as simple composition, strong glass-forming character, high thermal expansion coefficient, and low glass transition temperature^10,11^. Doping of these glasses with different agents expands their potential applications such as ionic and optoelectrical devices, and laser host materials^12^. The extraordinary physical properties of phosphate-based glasses such as low melting temperature, high conductivity to electricity, high thermal coefficient, and UV transmission give them scientific and technological importance^3,13–15^. The composite matrices fabricated from conductive polymers and glass were found to have desirable mixed candidates due to their wide range of applications, particularly as antimicrobial or biomaterial candidates^10–12^. The combination of polymers with glasses, ceramics, and glass ceramics can improve the workability and enhance the mechanical properties of the formed composites^16,17^. Recently, the antimicrobial activity for some phosphate glass/polyaniline composites in the presence of ZnO or CuO was reported, and the studied composites showed different responses to microorganisms. Their antimicrobial activity was found to be increased with increasing the content of CuO or ZnO and attained a maximum of 7 mol%^18^.

Now-a-days, there is a high demand for producing echo-friendly and nontoxic materials for every field application. Therefore, the combination of conducting polymers, phosphate glasses, and metal oxides, is quite popular, taking advantage of each component that helps produce a composite with excellent electrical performance that could be useful for electric energy storage applications. Therefore, the current article aims to fabricate and investigate the optical, electrical, and electrochemical properties of phosphate-based glasses (PBG) doped with metal oxides (CuO, ZnO) in the absence and existence of conductive polyaniline (PANI). Characterization of these composites was carried out using diffuse reflectance spectrophotometer (DRS), broadband dielectric spectrometer (BDS), cyclic voltammetry (CV), and charge–discharge technique. The combination of particular features leads us to determine the optimal use of these composites in the appropriate areas of application.

## Experimental procedure

### Samples preparation

The studied phosphate-based glasses (50 mol% P_2_O_5_—30 mol% K_2_O—(20-x) mol% CaO, where x is CuO or ZnO, were prepared from pure chemical materials. Ammonium dihydrogen orthophosphate (NH_4_H_2_PO_4_), anhydrous potassium carbonate (K_2_CO_3_), and calcium carbonate (CaCO_3_) were used as sources of P_2_O_5_, K_2_O, and CaO, respectively. However, copper and zinc ions were introduced as their respective oxides directly (CuO, ZnO). The composition of the prepared glasses and the codes used are listed in Table 1. In covered porcelain crucibles, the weighed batches were melted in an electric furnace (Vecstar, UK) at 500 °C to expel ammonia and water, after that, the temperature was raised and fixed at 1000 °C for 60 min with frequent rotation of the crucibles at intervals to promote homogeneity and complete mixing. The melts were poured on slightly warmed stainless steel molds and the prepared samples were immediately transferred to a muffle regulated at 285 °C. Then the annealing muffle was left to cool after 1 h to room temperature.

**Table 1:** Codes of the prepared samples.

PBG	(P_2_O_5_ 50 mol%–K_2_O 30 mol%–CaO 20 mol%)
3Zn/PBG	(P_2_O_5_ 50 mol%–K_2_O 30 mol%–CaO 17 mol%–ZnO 3mol%)
7Zn/PBG	(P_2_O_5_ 50 mol%–K_2_O 30 mol%–CaO 13 mol%–ZnO 7mol%)
3Cu/PBG	(P_2_O_5_ 50 mol%–K_2_O 30 mol%–CaO 17 mol%–CuO 3mol%)
7Cu/PBG	(P_2_O_5_ 50 mol%–K_2_O 30 mol%–CaO 13 mol%–CuO 7mol%)
BC	PBG/PANi
7Zn/BC	PBG/PANi/7Zn
7Cu/BC	PBG/PANi/7Cu
PANI	Polyaniline (PANi)

The polymer was prepared by oxidation of 5 ml of liquid aniline monomer in an acidic medium (HCl/1.25 M) by using potassium dichromate solution (K_2_Cr_2_O_7_/0.44 M) as an oxidant. The formed precipitate was washed with distilled water and dried in an autoclave at 70 °C for 2 h. To prepare the composite, 5 gm of the phosphate glass was ground to fine powder dissolved in distilled water and added to the polymerization bath.

The glasses and the glass/polymer composites as well as their surface morphology, structure, and crystalline structure were investigated in more detail in a recently published work^18^.

### Characterization

Different techniques were used to characterize the optical, electrical, and electrochemical properties of the prepared phosphate-based glasses.

#### Diffuse reflectance spectrometer (DRS)

The Diffuse reflectance spectrometer (DRS) is used to detect the direct and indirect forbidden band gap by measuring the diffuse reflectance in the wavelength range of 200–2500 nm through a double-beam spectrophotometer (JASCO: V-570 model).

#### Broadband dielectric spectrometer (BDS)

For the electric measurements, each sample was placed between the two electrodes of the measuring cell connected to a High-resolution impedance analyzer spectrometer (Schlumberger Solartron 1260). The measurements were carried out over a wide frequency range (10^–1^ to 10^6^ Hz) at room temperature.

#### Electrochemical studies

The electrochemical performance of phosphate-based glasses is performed by using Origalys OGS 200 potentiostat/galvanostat where the working electrode was prepared by mixing the active material, carbon black, PVDF (polyvinylidene fluoride) at the weight ratio of 80:10:10 using DMF (N, N-Dimethyl formamide) as a solvent to form a slurry and mixed by ultra-sonication for 30 min. Then 10μl of the suspension was dropped onto the surface of nickel foam with a micropipette, and then it dried at 60 °C for 40 min and then at room temperature overnight. Before that, the Ni foam was cleaned by degreasing in acetone, etching in 1 M HCl for 15 min, and subsequently washed with water and ethanol for 5 min each. The electrochemical investigations, such as cyclic voltammetry (CV) and galvanostatic charge–discharge (GCD), were carried out in a three-electrode conventional glass cell containing the electrolyte solution, which is 1.0 M KOH aqueous solution. The working electrode potential was measured against an Ag/AgCl reference electrode (*E*° = 0.203 V versus SHE), while pure Pt-wire was used as a counter electrode. Cyclic voltammetry curves were used to characterize the electrochemical behavior of our electrodes at different scan rates from 0.01 to 0.1V/s, covering a potential window (-0.5–1.0 V) (versus Ag/AgCl). Galvanostatic charge/discharge measurements were run in the potential window (−0.3 to 0.5 V) at current densities of 1, 3, 5, 8, and 10 A/g. All the previous methods were investigated in a recently published work^19^.

### Consent to participate

The authors agree to the journal’s policy.

## Results and discussion

### Optical properties

#### Theoretical optical basicity

In glass oxides, the identification of the negative charge that results from the presence of oxygen ions can be estimated by calculating the theoretical optical basicity $({\Lambda }_{{\text{th}}}$). It can be calculated by considering the individual optical basicity ${\Lambda }_{{\text{i}}}$ of each oxide and the proportion of oxygen atoms in the oxide ${\mathbf{X}}_{\mathbf{i}}$ according to the following equation^20^:

1 $${\Lambda }_{{\text{th}}} = \sum {{Z}_{i}{\text{X}}}_{{\text{i}}}{\Lambda }_{{\text{i}}}$$

where ${{\text{X}}}_{{\text{i}}}$,${Z}_{i,}$ and ${\Lambda }_{{\text{i}}}$ are the equivalent fractions, the valence cation, and the optical basicity of each oxide, respectively. For the studied glass system, it can be correlated as:

$${\Lambda }_{{\text{th}}/{\text{BC}}}=[\mathrm{X }\left({{\text{P}}}_{2}{{\text{O}}}_{5}\right)\Lambda \left({{\text{P}}}_{2}{{\text{O}}}_{5} \right)+\mathrm{ X }\left({{\text{K}}}_{2}O\right)\Lambda \left({{\text{K}}}_{2}O \right)+\mathrm{ X }\left({\text{CaO}}\right)\Lambda \left(\mathrm{CaO }\right)]$$

$${\Lambda }_{{\text{th}}/{\text{Zn}}}=[\mathrm{X }\left({{\text{P}}}_{2}{{\text{O}}}_{5}\right)\Lambda \left({{\text{P}}}_{2}{{\text{O}}}_{5} \right)+\mathrm{ X }\left({{\text{K}}}_{2}O\right)\Lambda \left({{\text{K}}}_{2}O \right)+\mathrm{ X }\left({\text{CaO}}\right)\Lambda \left(\mathrm{CaO }\right)+\mathrm{X }\left(ZnO\right)\Lambda \left(ZnO \right)]$$

$${\Lambda }_{{\text{th}}/{\text{Cu}}}=[\mathrm{X }\left({{\text{P}}}_{2}{{\text{O}}}_{5}\right)\Lambda \left({{\text{P}}}_{2}{{\text{O}}}_{5} \right)+\mathrm{ X }\left({{\text{K}}}_{2}O\right)\Lambda \left({{\text{K}}}_{2}O \right)+\mathrm{ X }\left({\text{CaO}}\right)\Lambda \left(\mathrm{CaO }\right)+\mathrm{X }\left(CuO\right)\Lambda \left(CuO \right)]$$

The values of optical basicity for ${{\text{P}}}_{2}{{\text{O}}}_{5}$, ${{\text{K}}}_{2}{\text{O}}$, CaO, ZnO, and CuO are 0.4, 1.4, 0.95, 1.04, and 1.03, respectively^20–22^. The calculated ${\Lambda }_{{\text{th}}}$ values listed in Table 2 show that doping with ZnO and CuO increases the theoretical optical basicity, which means an increase in the polarizability of the oxide ion (${\alpha }_{o}^{2-})$ as in the following equation:

**Table 2:** The theoretical optical basicity, direct and indirect band gap energy for some of the prepared glasses.

Sample	${\Lambda }_{th}$	E_g_ (direct), eV	E_g_ (indirect), eV
PBG	1.80	4.04	4.40
3Zn/PBG	1.80	3.80	3.80
7Zn/PBG	1.81	4.04	4.49
3Cu/PBG	1.80	4.28	4.76
7Cu/PBG	1.81	4.18	4.59

2 $${\Lambda }_{th}=1.67[1-\left(\frac{1}{{\alpha }_{o}^{2-}}\right)]$$

The increase of optical basicity values with doping is evidence of an increase in the ionic character of the glass samples doped with ZnO or CuO, which subsequently facilitates transferring electrons from the oxide ions to the cations surrounding it. Therefore, it is possible to use this kind of glass in the design of novel optical materials^23^.

#### Diffuse-reflectance spectra (DRS)

**Figure 1:**
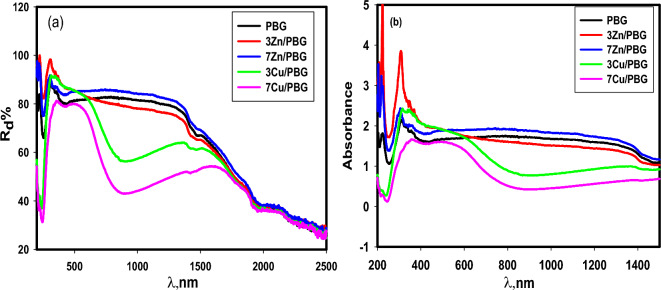
(**a**) The diffuse reflectance spectra, (**b**) the absorbance spectra of different glasses.

The diffuse-reflectance spectra of PBG, 3Zn/PBG, 7Zn/PBG, 3Cu/PBG, and 7Cu/PBG in the range of 200 to 2500 nm are illustrated in Fig. 1a. The reflectance increases suddenly at about 250 nm and then decreases slightly with increasing wavelength. Figure 1b represents the corresponding absorbance spectra, which show a sharp peak at about 304 nm for BC and Zn/BC while it broadens and appears at higher wavelength of 345–555 nm for Cu/BC due to the electronic transition of the d–d transition in the Cu ions^24^.

**Figure 2:**
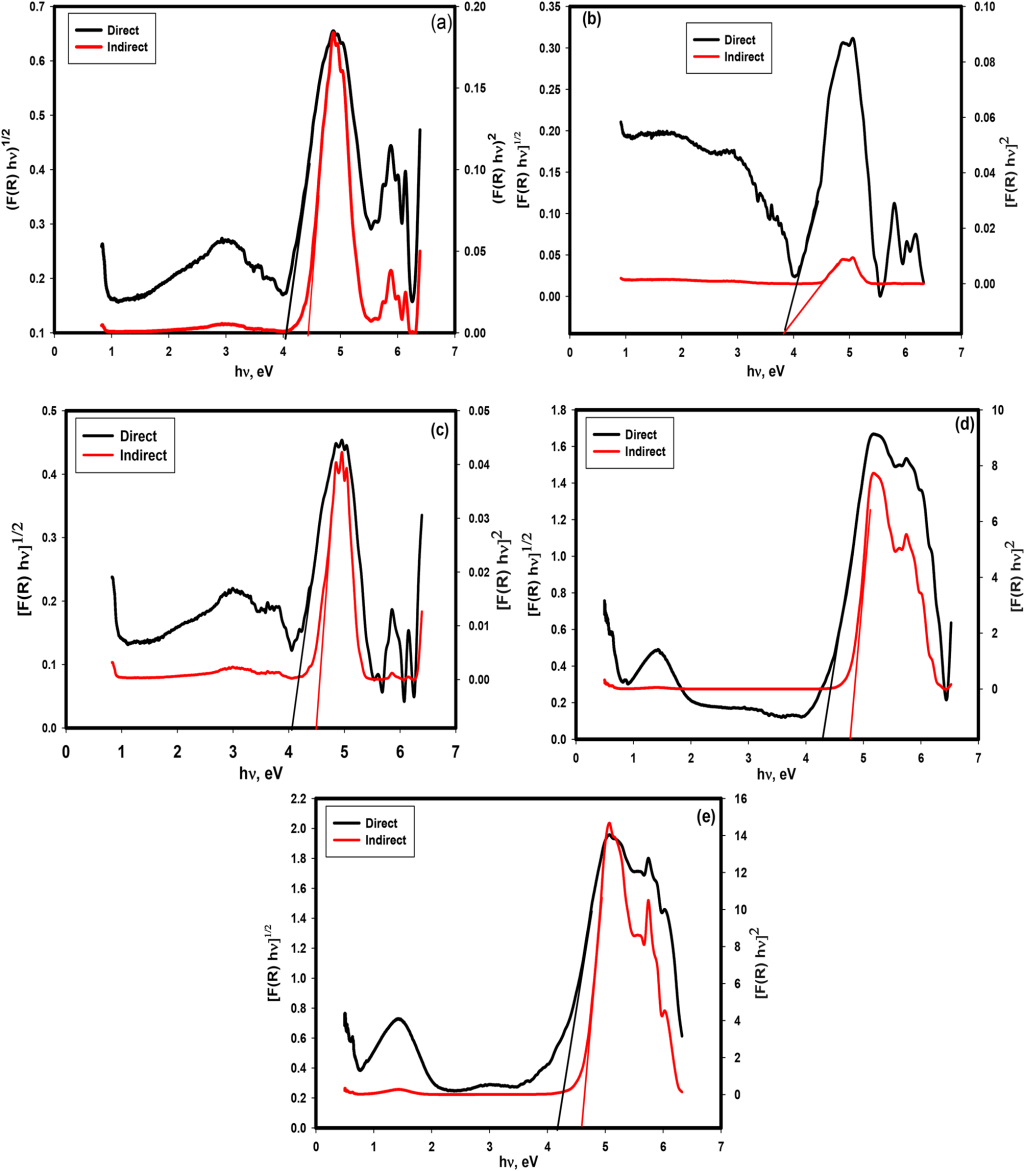
Tauc’s plots of different glasses (**a**) PBG, (**b**) 3Zn/PBG, (**c**) 7Zn/PBG, (**d**) 3Cu/PBG, and (**e**) 7Cu/PBG in the range of 200 to 2500 nm for direct and indirect cases.

The direct and indirect band gaps were evaluated for PBG, 3Zn/PBG, 7Zn/PBG, 3Cu/PBG, and 7Cu/PBG by using Tauc’s plots^19,25^ as in Fig. 2a–e. The direct and indirect band gaps for PBG, 3Zn/PBG, 7Zn/PBG, 3Cu/PBG, and 7Cu/PBG are presented in Table 2. Notably, the direct and indirect band gaps decreased by doping with ZnO while they increased by doping with CuO. The rise in the direct and indirect band gap values was related to the compactness of the network structure where increasing the number of bridging atoms leads to increasing the glass network compactness^22,26^. It was noted that the bandgap was widened by doping of ZnO and CuO on the PBG glass, which enhanced the transport of electrons and increased the growth of electroactive sites. This suggests the use of these materials for electronic and photo applications.

### Dielectric study

**Figure 3:**
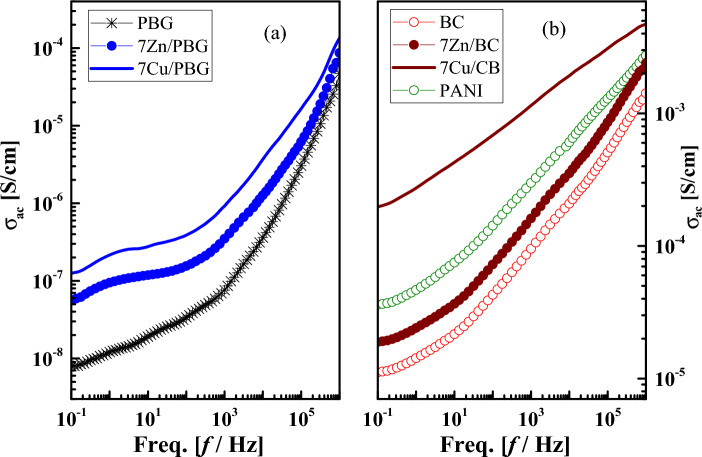
The frequency dependence of *ac* conductivity (*σ*_*ac*_) for (**a**) PBG, 7Zn/PBG, 7Cu/PBG, and (**b**) BC, 7Zn/BC, 7Cu/BC, and PANI.

**Figure 4:**
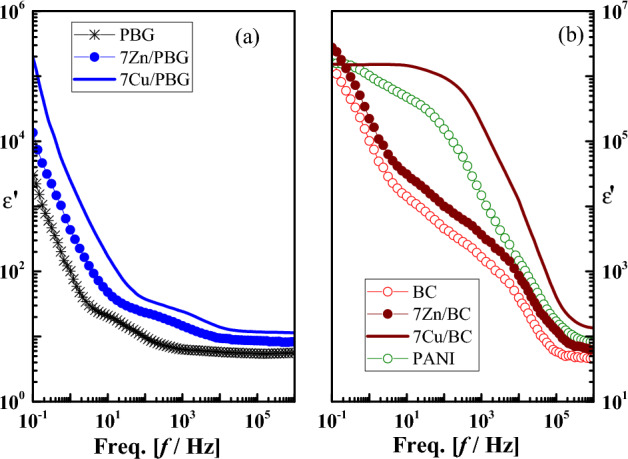
The frequency dependence of permittivity ($\varepsilon^{\prime}$) for (**a**) PBG, 7Zn/PBG, 7Cu/PBG, and (**b**) BC, 7Zn/BC, 7Cu/BC, and PANI.

**Figure 5:**
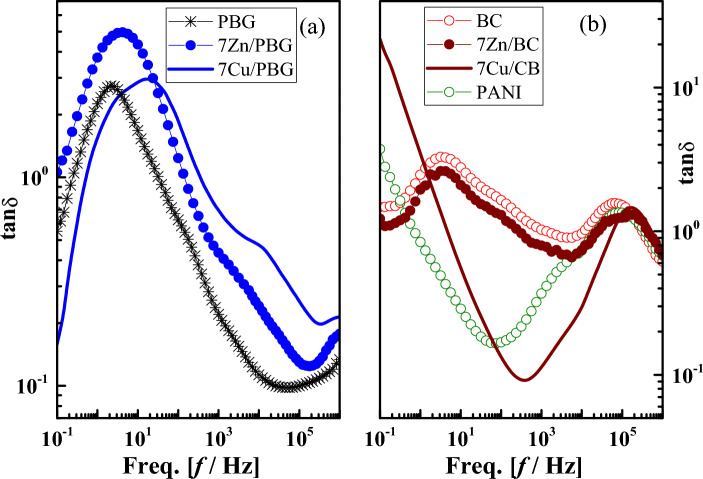
The frequency dependence of loss tangent (*tanδ*) for (**a**) PBG, 7Zn/PBG, 7Cu/PBG, and (**b**) BC, 7Zn/BC, 7Cu/BC, and PANI.

The dielectric properties (i.e. alternating-current conductivity *σ*_ac_, the permittivity $\varepsilon^{\prime}$, and the dielectric loss tangent *tanδ*, electric loss modulus *M''*) of the phosphate-based glasses (PBG) doped with metal oxides and mixed with polyaniline were firstly evaluated over a wide frequency range (10^–1^-10^6^ Hz) at room temperature. Although these properties are equivalent, i.e. directly related to each other, they provide different aspects of the underlying molecular dynamics and charge transport. The frequency dependencies of these properties are illustrated in Figs. 3, 4, and 5, respectively. Secondly, their glass composition dependence is evaluated at a constant frequency value (10^3^Hz) as listed in Table 3.

**Table 3:** The dielectric properties measured at 10^3^ Hz for the pure PANI, and different glass compositions.

Samples	*σ*_*ac*_ (S/cm)	$\varepsilon^{\prime}$	*tanδ*
Base Glass	7.6 × 10^–8^	1.4	0.22
7 Zn/PBG	3.5 × 10^–7^	15	0.43
7 Cu/PBG	9.2 × 10^–7^	24	0.7
BC	4.2 × 10^–5^	1664	1.02
7 Zn/BC	7.2 × 10^–5^	3655	0.8
7 Cu/BC	6.8 × 10^–4^	186,000	0.11
PANI	3 × 10^–4^	14,735	0.36

#### Frequency dependency

Alternating-current conductivity (σ_ac_) is an essential electrical property that determines the electrical conduction of a material upon applying an electrical field^27^. Furthermore, an increase in the concentration and mobility of ions leads to a conductivity increase^28^. The frequency dependence of AC conductivity (σ_ac_) is usually expressed by the Jonscher relation^29^:

3 $${\sigma }_{ac}=\sigma \left(\omega \right)-{\sigma }_{dc}=A{\omega }^{s}$$

where ω (= 2πf) is the angular frequency, A and s are constants, σ(ω) is the frequency-dependent conductivity measured under an AC field and σ_dc_ is the DC conductivity (σ_dc_). The frequency exponent (0 < s ≤ 1) is estimated from the slopes of the conductivity vs temperature plot (not presented here) and is used for determining the conduction mechanism^30–32^ as well as the modification of the glass network structure^33^. Generally speaking, the relationship (2) is valid for several low-mobility amorphous and even crystalline materials^34^. This relation was found to be valid for phosphate-based glasses (PBG) and those doped with 7 mol % metal oxides, i.e. 7Zn/PBG, and 7Cu/PBG, see Fig. 3a. For both glasses, σ_ac_ decreases towards frequencies lower than 1Hz, indicating the contribution of the electrode polarization (EP) phenomenon that arises from the building up of free charge carriers (electrons, ions, holes. etc.) on a macroscopic scale at the sample/electrode interface^35^. This phenomenon is manifested by the high values in both $\varepsilon^{\prime}$ (Fig. 4) and tanδ (Fig. 5). Further, the plateau-like behavior in conductivity within the frequency range (1–50 Hz) indicates the direct current or dc conductivity (σ_dc_) which arises from the random diffusion of the ionic charge carriers or/and jumping of ions to its neighboring vacant site^36^. At f > 50 Hz, the plateau-like behavior vanishes and the conductivity increases considerably, obeying the universal power law; σ_ac_ (ω) = Aω^s^. On the other hand, the remaining samples show a conductivity increase with low and high rates in the low and high -frequency regions, respectively. It can also be noticed that the conductivity of most glass compositions, particularly Zn/PBG and Cu/PBG, have semiconducting features at f ≥ 10^3^ Hz.

The permittivity or the real part of complex permittivity ($\varepsilon^{\prime}$) measures the energy stored from the applied electric field in the material and identifies the strength of alignment of dipoles in the dielectric. It is a function of an electrical capacitance (C) and is given as:

4 $$\varepsilon^{\prime} (\omega )=C(\omega )\frac{d}{{\varepsilon }_{o}A}$$

where ω is the angular frequency (= 2πf) where f is the frequency of the applied electric field in Hertz and ε_o_ = 8.85 × 10^–12^ F/m is the vacuum permittivity. The sample geometry is denoted as d (thickness) and A (sample surface area). In Fig. 4, the permittivity ($\varepsilon^{\prime}$) of all glass compositions shows high values due to the contribution of all polarization components^37,38^, causing an overall polarization increase^39^. In the present study, two polarization mechanisms, i.e. space charge (Ps) and dipolar (Pd) dominate the dielectric properties of the glass compositions at low (up to 10^3^Hz) and higher frequency (up to ~ 10^10^Hz), respectively. Ps arises from the separation of charge carriers at interfaces. When the charge carriers separate on a macroscopic scale at the sample/electrode interface, an electrode polarization is obtained which displays extremely high values in both $\varepsilon^{\prime}$ and dielectric loss ($\varepsilon^{\prime\prime}$ = $\varepsilon^{\prime}$ × tanδ). When the charge carriers separate on a microscopic scale at the internal layers or interfaces of an inhomogeneous system, an interfacial or Maxwell–Wagner Sillars (MWS) polarization is obtained. The dipolar polarization (Pd) arises from the orientation of the electric dipole moment towards an electric field. The observed permittivity decrease with frequency is due to a decrease in overall polarization. This can also be explained based on the well-known dielectric relaxation phenomenon which suggests that the charge carrier localization is unstable and easily affected by the frequency of the electric field^40^.

The permittivity of BC and 7Zn/BC samples (Fig. 4b) show typical permittivity decreases with frequency. It shows a frequency independence or a plateau-like behavior at f < 10 Hz for 7Cu/BC which could be possibly due to the coexistence of electrode polarization and DC conductivity. Then, it decreases sharply at f > 10^3^ Hz, indicating the predominance of dipolar polarization. From above, the conductivity, space charge, and dipolar polarization may dominate the dielectric behavior of the presented samples.

Compared to the permittivity ($\varepsilon^{\prime}$), the dielectric loss tangent loss (tanδ) measures the energy dissipated in the dielectric materials that are associated with the frictional dampening, which prevents the displacement of bound charge from keeping in phase with the field change. Its relationship with dielectric loss ($\varepsilon^{\prime\prime}$) and permittivity ($\varepsilon^{\prime}$) is given as:

5 $$tan\delta \left(\omega \right)=\frac{\varepsilon ^{\prime\prime} \left(\omega \right)}{\varepsilon^{\prime} \left(\omega \right)}$$

Figure 5 shows the frequency-dependent tanδ of PBG, 7Zn/PBG, 7Cu/PBG, BC (PBG/PANI), 7Zn/BC, 7Cu/BC, and PANI. So, the loss spectra of all samples except BC and 7Zn/BC, show a low-frequency relaxation peak (LFRP) connected to a shoulder. The average peak width is higher than the Debye peak width (1.14 orders)^41^, due to the distribution of relaxation times. It is believed in dielectric analysis that LFRP is mainly attributed to the contributions of interfacial polarization, or/and DC conductivity. Interfacial or Maxwell Wagner Sillar (MWS) polarization arises mainly from the existence of polar and conductive regions dispersed in a relatively less polar and insulating matrix^37,38^. Because of this, one can attribute LFRP to the building up of free ions (Cu or Zn) at interfaces between metal oxides (ZnO or CuO) and PBG due to the conductive difference between them. This peak shifts to higher positions upon doping with metal oxides and/or mixing with PANI, indicating enhancement of mobility and/or concentration of free ions, i.e. a conductivity increase^37^. As a representative example, the low-frequency relaxation peak position of the pure PBG (~ 2 Hz) shifts to 4 Hz and 18 Hz upon doping with ZnO and CuO, respectively, due to the coexistence of electrode polarization and DC conductivity. On the other hand, the high-frequency relaxation peak (HFRP) positioned at ~ 10^5^ Hz of BC, PANI, 7Zn/BC, and 7Cu/BC could correspond to the dipolar polarization, i.e. rotation of the amino group (NH_2_). Compared to all the glass compositions, the dielectric loss tangent (tanδ) for both PANI and 7Cu/BC sharply decreases with increasing frequency up to 56 and 317 Hz, respectively. One may attribute this behavior to the DC conductivity contribution.

**Figure 6:**
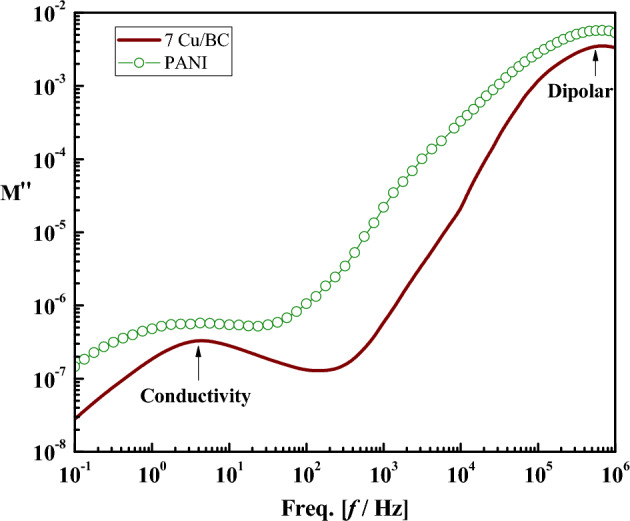
The frequency dependence of the electric loss modulus for PBG, 7Zn/PBG, 7Cu/PBG, BC, 7Zn/BC, 7Cu/BC, and PANI.

To understand deeply the relaxation process in the low-frequency region, the frequency dependence of electric loss modulus ($\text{M}^{\prime\prime}=\varepsilon^{\prime\prime} / \varepsilon^{\prime{2}} + \varepsilon^{\prime\prime{2}}$) instead of tanδ is recommended, see Fig. 6. This is because M''-f representation is useful to suppress the capacitance effects of electrode polarization and provide a clear view DC conduction and dipole relaxation^42,43^. From the figure, M'' shows a very small value ≈ 0, indicating the absence of electrode polarization effect. Further, it shows a new relaxation peak in the low-frequency region whose maximum is positioned at the same frequency value (~ 4 Hz) for both PANI and 7Cu/BC. According to the Kramers–Kronig relationship^44^ the appearance of such a peak confirms the contribution of DC conductivity.

#### The glass compositions dependent electric properties

To understand deeply the effect of PANI, ZnO, and CuO on the dielectric properties of phosphate-based glasses (PBG), the electrical properties of all samples measured at 10^3^ Hz are summarized in Table 3. As clear, the pure PBG exhibits the lowest electric properties (σ_ac_ = 7.6 × 10^–8^ S/cm, $\varepsilon^{\prime}$ = 1.4 and tanδ = 0.22) compared to the remaining glass samples due to its insulating nature. When it is doped with the semiconducting metal oxides (ZnO or CuO), its properties show an increase due to a polarizability increase that agrees with the theoretical optical basicity increase mentioned above ("Theoretical optical basicity"). Further, in doping with these oxides, the bandgap is widened, causing an enhancement of electronic transportation which in turn increases the growth of electroactive sites. One can also notice that the electrical properties are higher in CuO-doped glasses than in ZnO-doped glasses. This behavior could be related to the changes in the direct and indirect band gap as reported by the optical properties mentioned above in "Diffuse-reflectance spectra (DRS)". So, the direct and indirect band gap was found to have higher values in CuO-doped glasses than in ZnO-doped glasses. In addition, Copper is a semiconducting transition metal, its ions exist in two valence states (Cu^+1^, Cu^+2^), through which the electrical conduction occurs by hopping of polarons from ions of a lower valence state (Cu^+1^) to ions of a higher valence state (Cu^+2^)^45,46^. These states encourage the replacement of P–O–P bonds with P–O–Cu^+^ or P–O–Cu^2+^ bonds, which significantly improves the conductivity and electric properties of CuO-based composites Cu/PBG^47,48^. Further, doping with CuO leads to the existence of mixed electronic–ionic electrical conduction. Another advantage of CuO over ZnO is Cu^+^ ions incorporated or housed into the glass network, which in turn expands into voids, reducing the pathways in the network. Consequently, the activation energy became lower, causing the mobile Cu^+^ ions to migrate easily^49,50^. Besides, the electrical properties of glasses were found to be highly enhanced upon mixing with polyaniline (PANI), particularly for those doped with CuO. For instance, 7Cu/BC shows a semiconducting feature (*σ*_*ac*_ = 6.8 × 10^–4^ S/cm), giant $\varepsilon^{\prime}$ (186,000), and lower *tanδ* = 0.11. These values are common in ionic conducting materials in which the mobile ions reach both electrodes at very low frequencies, making a thin and poor conducting space charge region, which acts as massive capacitors. It is worth mentioning that the magnitude of the room temperature permittivity of this glass sample demonstrates the potential advantage for energy storage over pure PBG (ε ~ 1.4).

### The electrochemical studies

**Figure 7:**
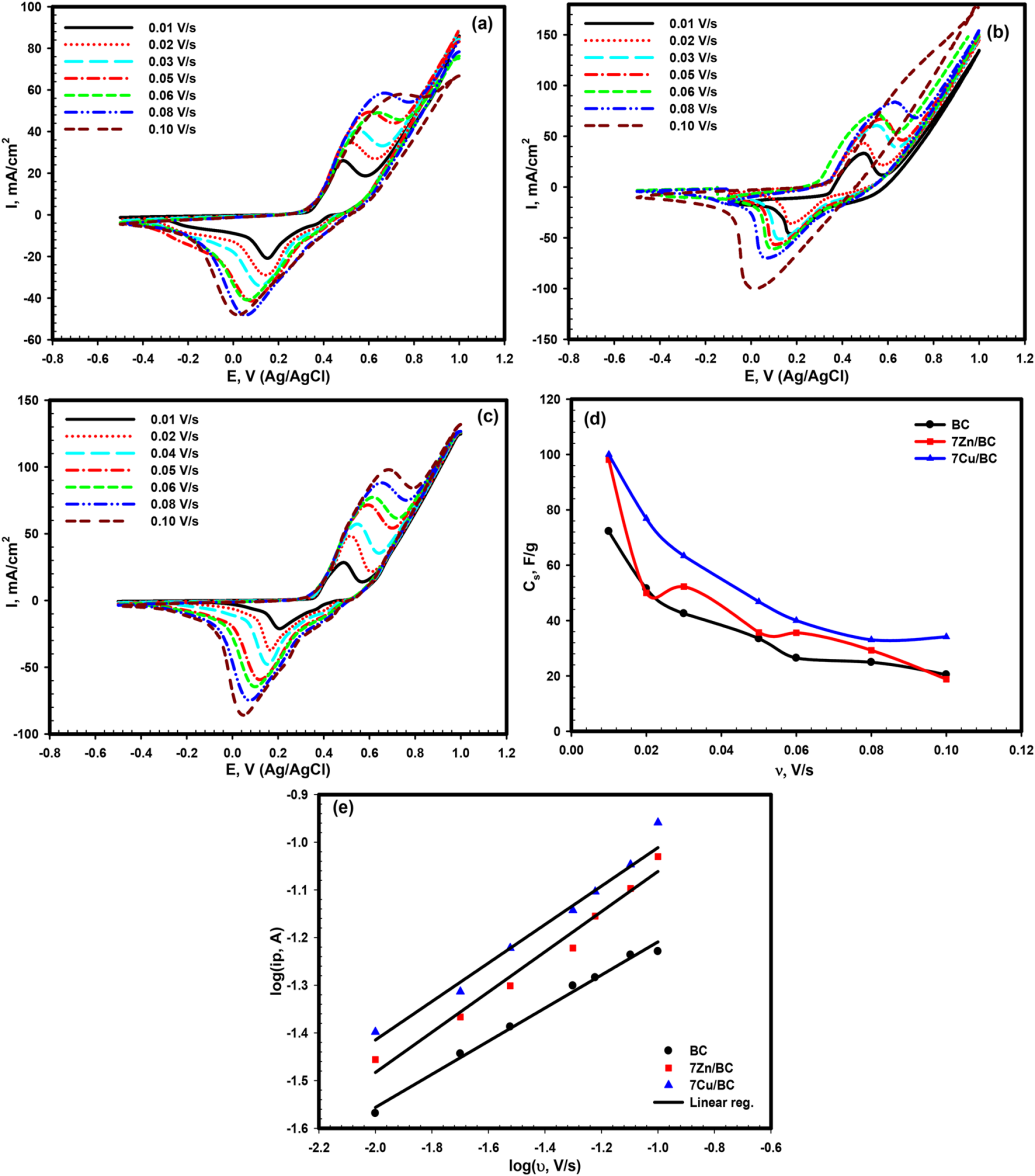
Cyclic voltammetry for (**a**) BC, (**b**) 7Zn/BC, (**c**) 7Cu/BC in 1.0 M KOH aqueous solution at different scan rates (0.01–0.1 V/s), (**d**) the relation between the scan rate and the specific capacitance for the three samples, (**e**) the relation between logarithm scan rate and logarithm current for the three samples.

The cyclic voltammetry graphs for BC, 7Zn/BC, and 7Cu/BC in 1.0 M KOH aqueous solution using Ag/AgCl as a reference electrode at different scan rates (0.01–0.1 V/s) over the potential window (−0.5–1.0 V) were observed in Fig. 7a–c. The CV curves for BC, 7Zn/BC, and 7Cu/BC at scan rate 0.01 V/s (Fig. 7a–c) show one oxidation peak at potential 0.48 V, and the corresponding reduction peak at 0.15 V, the redox peak is due to the redox reaction of polyaniline from leucoemeraldine to the emeraldine^51^. The behavior of the CV curves for the samples indicates the pseudo-capacitive behavior. A shift in the anodic and cathodic peaks (*i*_*p*_) is observed for the three samples to more positive and negative potential values, respectively, as the scan rate ($\upsilon$) increases which indicates low resistance of the materials and the acceleration in the diffusion of ions^52^. Furthermore, the peak current values also increase with increasing the scan rate and accordingly, the area under the CV curves was enlarged due to the improvement in the transfer of the electron through the material.

The specific capacitance (*C*_*s*_) of BC, 7Zn/BC, and 7Cu/Bc was calculated with the aid of the area under the CV curve $(\int IdV)$, using the following relation,

6 $${C}_{s}= \frac{\int IdV}{2m\upsilon \Delta V}$$

where $\Delta V$ is the potential window (−0.5 to 1.0 V), *m* is the active mass of the material in gm and $\upsilon$ is the scan rate (V/s). The relation between the calculated specific capacitance and the scan rate is shown in Fig. 7d, where a reduction in the value of the specific capacitance occurs with increasing the scan rate because of the decrease in the active sites with decreasing the scan rate where there is enough time for the ions to get up at the surface of the electrode and to interact with it^19,53,54^. It was noted from Fig. 7d that the values of C_s_ for 7Cu/CB at nearly all the scan rates are higher than C_s_ for CB and 7Zn/CB, since the highest value for 7Cu/CB is 100 F/g at a scan rate of 0.01 V/s. The ion transfer mechanism for the studied materials (BC, 7Zn/BC, and 7Cu/Bc) can be estimated from the linear relation between log ${i}_{p}$ and $log \upsilon$ for both the anodic and cathodic peaks according to the following equation:

7 $${\text{log}}{i}_{p}={\text{log}}a+b log \upsilon$$

The slope of the straight line was represented by b (Fig. 7e), and the value of b can help in suggesting the mechanism of ion transfer. When b is close to or equal to 0.5, the ion transfer was suggested to be diffusion controlled and the capacitance behavior of the material is like battery type-one, and when it is equal to or close to 1.0, the mechanism was controlled by surface process and the material showed capacitive type nature. The value of b for BC, 7Zn/BC, and 7Cu/BC is close to 0.5, which suggests the battery type-one nature of the three materials, and the ion transfer occurs through a diffusion- controlled mechanism^55,56^.

**Figure 8:**
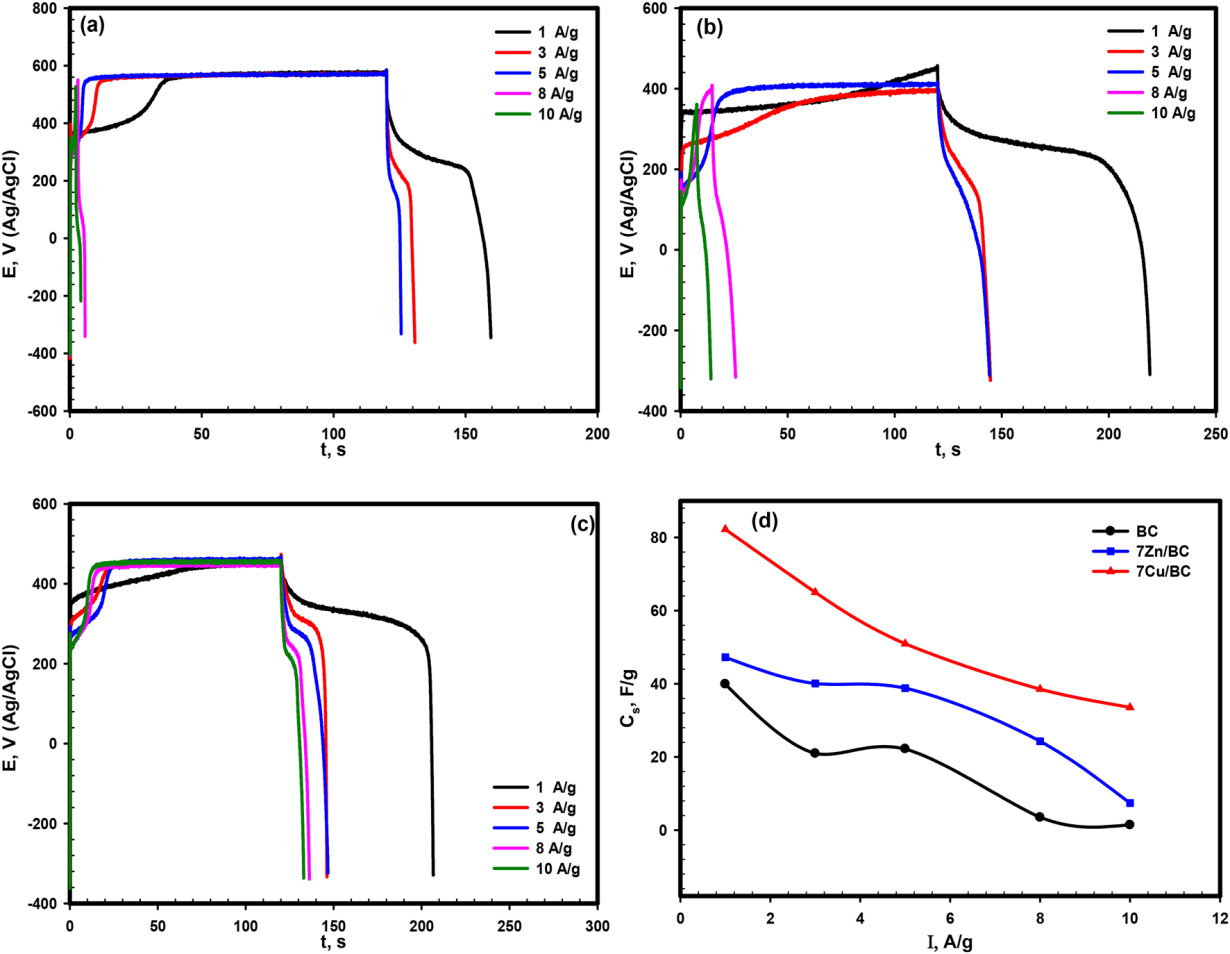
Galvanostatic charge–discharge curves in 1.0 M KOH (**a**) BC, (**b**) 7Zn/BC, and (**c**) 7Cu/BC at various current densities and (**d**) the relation between the applied current density and the specific capacitance for the three samples.

The galvanostatic charge–discharge curves conducted for BC, 7Zn/BC, and 7Cu/BC in a potential range of −0.3 to 0.6 V (vs. Ag/AgCl) in 1.0 M KOH at different current densities (1–10 A/g) as shown in Fig. 8a–c where the curves are nonlinear, which confirms the pseudocapacitive behavior (faradic) as in CV measurement. Therefore, the specific capacitances of the studied materials were calculated via the following equation by integrating the area under the discharge curve($\int V(t)dt$ )^55^:

8 $${C}_{s}=\frac{I\int V(t)dt}{m (\Delta V{)}^{2}}$$

where m is the active mass, I; is the current density, and the difference in the potential window. Figure 8d shows the effect of the applied current densities on the specific capacitance values. It is noted that applying a large current results in decreasing the specific capacitance values due to the decrease in the ions of the electrolyte at the surface active area because the time is insufficient at high current density^57^. At a current density value of 1.0 A/g, the highest value of specific capacitance is 82.3 F/g. The value of the specific capacitance for 7Cu/BC is larger than BC and 7Zn/BC because of the improvement in both conductivity and dielectric properties by doping with CuO, as the values of ac-conductivity, dielectric constant and dielectric loss for 7Cu/BC are common in ionic conducting materials as illustrated in the electrical studies section. In addition, the presence of two valence states (Cu^+1^, Cu^+2^) leads to the hopping of polarons from ions of a lower valence state (Cu^+1^) to ions of a higher valence state (Cu^+2^)^45,46^. Also, the presence of PANI with the metal oxide enhanced the mobility and/or concentration of free ions in the composite^37^.

**Figure 9:**
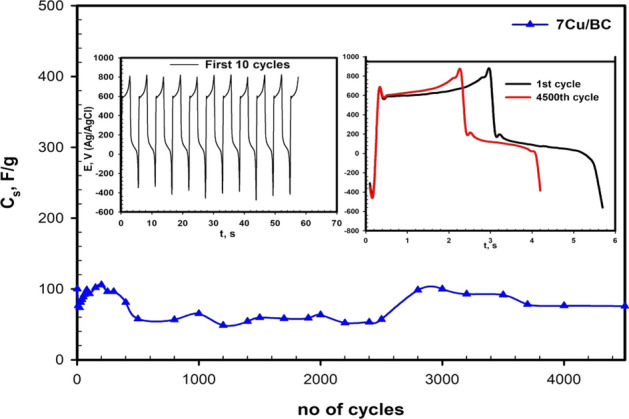
Charge–discharge cycling stability curve for 7Cu/BC at a current density of 8 A/g (the inset figures is for the first 10 cycle and the charge–discharge of the first and the 4500 the cycle).

Long-term cycling is important in showing the stability of the materials used as supercapacitors. Therefore, 4500 charge–discharge cycles for 7Cu/CB at 8 A/g were performed in the potential windows (-0.3 to 0.8 V). Figure 9 shows the capacitance retention change with the number of cycles slightly. It is noted an increase in the specific capacitance during the first 200 cycles to 113% for the first cycle, then decreased to about 55% after 2500 cycles and increased again to reach 77% after 4500 cycles, indicating good stability of 7Cu/BC. The increase in the retention is due to increasing the electrolyte temperature with increasing the time of operation^58^.

## Conclusion

In the current study, the electrical, optical, and electrochemical behavior of the phosphate-based glasses (PBG), containing ZnO or CuO in the absence and existence of conductive polyaniline (PANI). The glass and the glass doped with 3ZnO, 7ZnO, 3CuO, and 7CuO exhibit both direct and indirect transition with values of transition gap of (4.04 and 4.4), (3.8 and 3.8), (4.04 and 4.49), (4.28 and 4.76) and (4.18 and 4.59), respectively. The dielectric measurements were carried out over a wide frequency range at room temperature. The dielectric properties of PBG have been enhanced upon doping with metal oxides and/or mixing with PANI. Particularly, the *AC* conductivity of PBG doped with 7 mol% CuO and mixed with PANI exhibited a semiconducting feature (6.8 × 10^–4^ S/cm), lower dielectric loss tangent (0.11), and giant permittivity (186,000). The cyclic voltammetry of the studied composites shows one redox couple that demonstrates the pseudo-capacitive behavior of the material. The reaction through the studied materials was diffusion-controlled, and the material exhibited battery type-one in nature. The highest value of the specific capacitance is 82.3 F/g at 1.0 A/g for 7Cu/BC, and the initial specific capacitance was increased to about 113% in its first cycle, then decreased to about 55% after 2500 cycles, and increased again to reach 77% after 4500 cycles indicating good stability of 7Cu/BC. The combination of optical, electrical, and electrochemical features of these composites leads to several unique advantages for energy generation and/or storage devices.

## Data Availability

Data will be available from the corresponding author upon reasonable request.
